# Integrative analyses of biomarkers and pathways for diabetic nephropathy

**DOI:** 10.3389/fgene.2023.1128136

**Published:** 2023-04-11

**Authors:** Bo Li, Xu Zhao, Wanrun Xie, Zhenzhen Hong, Yi Zhang

**Affiliations:** ^1^ Department of Endocrinology, Quanzhou First Hospital Affiliated to Fujian Medical University, Quanzhou, China; ^2^ Emergency and Critical Care Center, Renmin Hospital, Hubei University of Medicine, Shiyan, China

**Keywords:** bioinformatics, biomarkers, diabetic nephropathy, bioinformatics analysis, enrichment analysis

## Abstract

**Background:** Diabetic nephropathy (DN) is a widespread diabetic complication and a major cause of terminal kidney disease. There is no doubt that DN is a chronic disease that imposes substantial health and economic burdens on the world’s populations. By now, several important and exciting advances have been made in research on etiopathogenesis. Therefore, the genetic mechanisms underlying these effects remain unknown.

**Methods:** The GSE30122, GSE30528, and GSE30529 microarray datasets were downloaded from the Gene Expression Omnibus database (GEO). Analyses of differentially expressed genes (DEGs), enrichment of gene ontology (GO), the Kyoto Encyclopedia of Genes and Genomes (KEGG), and gene set enrichment analysis (GSEA) were performed. Protein-protein interaction (PPI) network construction was completed by the STRING database. Hub genes were identified by Cytoscape software, and common hub genes were identified by taking intersection sets. The diagnostic value of common hub genes was then predicted in the GSE30529 and GSE30528 datasets. Further analysis was carried out on the modules to identify transcription factors and miRNA networks. As well, a comparative toxicogenomics database was used to assess interactions between potential key genes and diseases associated upstream of DN.

**Results:** Samples from 19 DNs and 50 normal controls were identified in the GSE30122 dataset. 86 upregulated genes and 34 downregulated genes (a total of 120 DEGs). GO analysis showed significant enrichment in humoral immune response, protein activation cascade, complement activation, extracellular matrix, glycosaminoglycan binding, and antigen binding. KEGG analysis showed significant enrichment in complement and coagulation cascades, phagosomes, the Rap1 signaling pathway, the PI3K-Akt signaling pathway, and infection. GSEA was mainly enriched in the TYROBP causal network, the inflammatory response pathway, chemokine receptor binding, the interferon signaling pathway, ECM receptor interaction, and the integrin 1 pathway. Meanwhile, mRNA-miRNA and mRNA-TF networks were constructed for common hub genes. Nine pivotal genes were identified by taking the intersection. After validating the expression differences and diagnostic values of the GSE30528 and GSE30529 datasets, eight pivotal genes (TYROBP, ITGB2, CD53, IL10RA, LAPTM5, CD48, C1QA, and IRF8) were finally identified as having diagnostic values.

**Conclusion:** Pathway enrichment analysis scores provide insight into the genetic phenotype and may propose molecular mechanisms of DN. The target genes TYROBP, ITGB2, CD53, IL10RA, LAPTM5, CD48, C1QA, and IRF8 are promising new targets for DN. SPI1, HIF1A, STAT1, KLF5, RUNX1, MBD1, SP1, and WT1 may be involved in the regulatory mechanisms of DN development. Our study may provide a potential biomarker or therapeutic locus for the study of DN.

## Introduction

The worldwide prevalence of diabetes continues to increase dramatically and is predicted to rise to nearly seven hundred million in 2045 (Cho et al., 2018). The leading cause of both chronic kidney disease (CKD) and end-stage renal disease (ESRD) is diabetes mellitus (GBD Chronic Kidney Disease Collaboration, 2020). DN affects a high proportion of diabetics worldwide and is a microvascular disease (Wang et al., 2021). The commonest reason for end-stage chronic kidney disease is DN, and it has a serious impact on the quality of life of patients (Liu et al., 2021). Compared with non-DN patients, urinary protein levels were significantly higher in DN patients (*p* < 0.001), and mitochondria in podocytes were more fragmented in DN patients than in non-DN patients (Ma et al., 2019). The early appearance of microalbuminuria in DN should be screened at an early stage and followed up regularly (Bakris and Molitch, 2014). DN accompanies 40% of patients with diabetes and is related to considerable morbidity and mortality (Macisaac et al., 2014).

DN progresses through normoalbuminuria, microalbuminuria, or early DN, macroalbuminuria, and ultimately to ESRD (Sourris and Forbes, 2009). The hallmark indicators of renal function are the estimated glomerular filtration rate (eGFR) and albuminuria (Persson and Rossing, 2018). But these do not provide advance warning of a DN. Through research, questions have been raised about their reliability as DN diagnostics (Macisaac et al., 2014; Bjornstad et al., 2015). It has now been discovered that DN can progress directly to ESRD without albuminuria, which challenges the diagnostic value of albuminuria (MacIsaac and Jerums, 2011; MacIsaac and Ekinci, 2019). It is common practice to detect microalbuminuria at the early stages of DN; However, some patients with microalbuminuria have advanced renal disease. Microalbuminuria is influenced by many factors, and its reliability and accuracy are disputed (Zhou et al., 2021). At the same time, eGFR does not exactly reflect measured GFR (mGFR), which could lead to an underlying misclassification of renal function (MacIsaac et al., 2015). Serum creatinine has been questioned as a marker as well, so there is an urgent need to find reliable biomarkers to predict ND occurrence and progression (Colhoun and Marcovecchio, 2018).

The DN treatment is not very effective, and the cost of its treatment is consistently a significant expense in any country. Early diagnosis of diabetic nephropathy is important for early intervention and treatment. With the speedy advancement of sequencing technology, a variety of research associated with the pathophysiological course of DN has been conducted, and an increasing number of new biomarkers have been identified (Fan and Hu, 2022). These biomarkers have been shown to be associated with the inflammatory and renal injury pathways of DN, as well as with eGFR and albuminuria, increasing their predictive and diagnostic properties (Colhoun and Marcovecchio, 2018). TNFR1, CRP, TNF-, CCL15, Glypican-5, MMPs, and VEGF are a few examples. However, there is still a deficit in clinical evidence. In the absence of symptoms or early symptoms, the expression levels of relevant biological signaling molecules, cytokines, and other substances may already have changed. Therefore, further research on the molecular mechanisms, such as cytokines, involved in the progression of DN is required to explore more DN-related biomarkers and improve their relevant clinical evidence, thus improving the early diagnosis and prognostic management of DN for the benefit of patients.

The global prevalence of DN is currently a significant public health concern. It is required to investigate potential biomarkers and molecular pathways linked to the onset and progression of DN. Bioinformatics has become a critical technique for elucidating the pathogenesis, etiology, and therapy of DN.

In this study, we chose the GSE30122, GSE30528, and GSE30529 datasets of the platform GPL571 from the Gene Expression Omnibus (GEO), which is the transcriptome analysis of human diabetic kidney disease. Identify the potential DEGs that participate in the initiation and development of DN and analyze their expression, function, and interaction in order to serve as a guide for researching potential biomarkers or therapeutic targets for DN.

## Methous

### Data acquisition

Screen potential diabetic nephropathy-related genes using GEO datasets and text mining. The transcriptome expression profile datasets GSE30122, GSE30529, and GSE30528 (Table 1) were obtained on the GPL571 platform of the GEO database. The datasets were composed of normal control samples and diabetic nephropathy samples. Lastly, 19 DN and 50 normal group samples were analyzed in the GSE30122 dataset. Meanwhile, GSE30528 and GSE30529 were used for further screening of the key genes and to probe the expression of common key genes in the GSE30528 dataset. Statistical analysis was performed by quantile-normalizing and log2-transforming the raw data.

**TABLE 1:** Details of GEO DN data in this study.

Accession	Platform	Tissue	Tissue Subregion	control	DN	Gene
GSE30122	GPL571	kidney	glomerulus+tubules	50	19	mRNA
GSE30528	GPL571	kidney	glomerulus	13	9	mRNA
GSE30529	GPL571	kidney	tubules	12	10	mRNA

*GSE, gene expression omnibus; DN, diabetic nephropathy.

**FIGURE 1:**
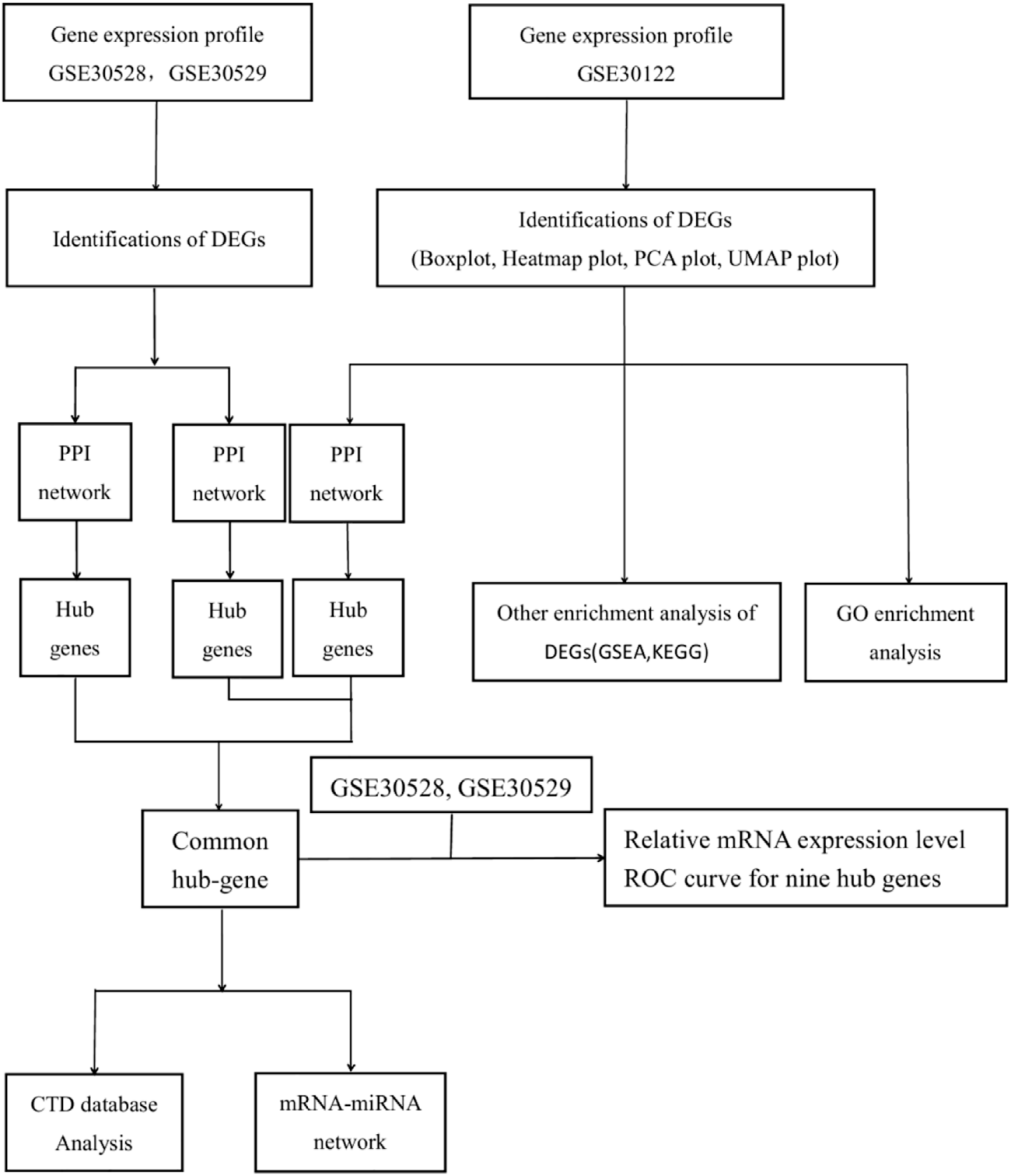
Presents the flowchart of the study.

The details of the three datasets are shown in Table 1, and the flowchart of the study is shown in Figure 1.

### Analysis of differential gene expression

These datasets were downloaded from the GEO database, and only the probes with the highest signal values for the same molecule were retained. The Limma software package was again used to normalize the data and analyze the variance between the two groups.

DEGs were calculated using the R software package “limma” (Davis and. Meltzer, 2007), with *p* values adjusted < 0.05 and |log2FoldChange| > 1. The “Complex Heatmap” (version 2.2.0) and “ggplot2” packages (version 3.3.3) of R software (version 3.6.3) were used to create volcano maps, PCA maps, and heat maps. (Gu et al., 2016).

### GO and KEGG pathway analysis

To conduct GO and KEGG pathway analysis, the ClusterProfiler package (version 3.14.3) of R software was used (Yu et al., 2012). The org.hs.eg.db package (version 3.10.0) for ID conversion, and the GOplot package (version 1.0.2) for calculating the z-score (Walter et al., 2015). The adjusted *p*-value was 0.05 as a measure of statistical significance. The GO enrichment analysis included biological process (BP), cellular component (CC), and molecular function (MF) (www.frontiersin.org).

### Gene set enrichment analysis (GSEA)

GSEA was performed in order to explore biological signaling pathways. When the FDR <0.25 and the p. adjust value <0.05, it is thought to be a significant enrichment and is used as a screening index. Mainly, the clusterProfiler package (version 3.14.3) was used for GSEA analysis (Yu et al., 2012). Statistical analysis and visualization using R software (version 3.6.3).

### Protein-protein interaction network analysis

The STRING database (https://string-db.org/) was used to construct the PPI network to reveal general organizational principles of cellular function and predict protein-protein interactions (Damian et al., 2018), perform modular analysis, and visualize the results of the PPI network through the MCODE of Cytoscape (version 3.9.1). Using the Cytohubba plugin in Cytoscape, the 20 highest-scoring genes were labeled as “hub genes” using the MCC algorithm in Cytoscape. The hub genes of the three datasets were used as an intersection and as a common hub gene for the validation analysis.

### Structure of mRNA-miRNA and mRNA-TF modulatory networks

Prediction of interactions with the miRNet database 2.0 between differentially expressed miRNAs and mRNAs (prediction URL: https://www.mirnet.ca/). Then, the mRNA-miRNA regulatory networks and the mRNA-TF regulatory networks were constructed to profile the interactions with mRNAs and miRNAs/TF as target potential for DN renal cells. The regulatory network was visualized using Cytoscape software.

### Validation of common hub genes

The R package partial (pROC) was used for receiver operating characteristic (ROC) curve analysis and computation of ROC curves and ROC AUC values. Visualization of charts is implemented with the ggplot2 package. Multi-gene ROC analysis is a predictor of probability based on the contribution of multiple genes to the outcome. A ROC analysis was performed on the results of binary logistic regression calculations for each sample. Regression was performed with the SPSS 22.0 version. Outcomes were quantified as the area under the ROC curve (AUC) of the results, and the genes with AUC > 0.7 were deemed diagnostic.

### Identification of key potential genes related to DN

The Comparative Toxicogenomics Database (CTD, http://ctdbase.org/, accessed December 10, 2022) is an integrated database that integrates information related to chemical gene-protein interactions, chemical disease, and genetic disease relationships and proposes postulates associated with disease mechanisms (Davis et al., 2018). Data from the CTD were used to characterize the relationship of potential key genes to diseases upstream of the DN, such as insulin resistance, diabetes, metabolic syndrome, hyperlipidemia, and acidosis.

### Statistical analysis

We used R software v. 3.6.3 for strategic analysis. Figures were presented in terms of means and standard deviations, and comparisons between groups were made using unpaired t-tests. A *p*-value < 0.05 was considered statistically significant.

## Result

### Expression profiling data

**FIGURE 2:**
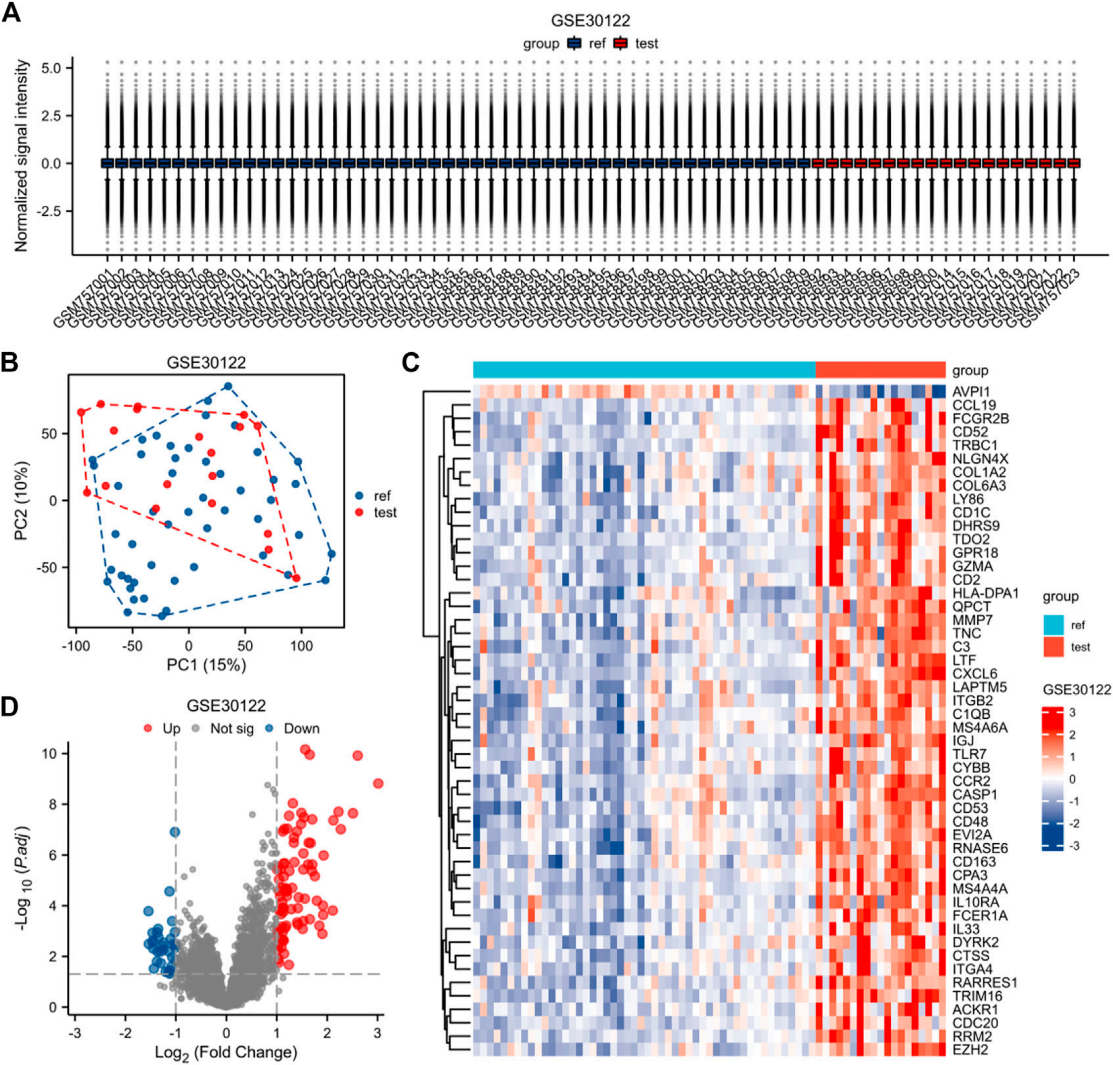
Normalized expression matrices **(A)** of the GSE30122 dataset. Differentially expressed genes from the GSE30122 dataset using a |log2 FC|1 screening criterion and an adjusted *p*-value of 0.05 **(B–D)**. [**(B)** PCA plot; **(C)** heatmap plot; **(D)** Volcano plots in GSE30122. PCA: Principal Component Analysis; Ref: Control Group; Test: Diabetic Nephropathy, DN].

**FIGURE 3:**
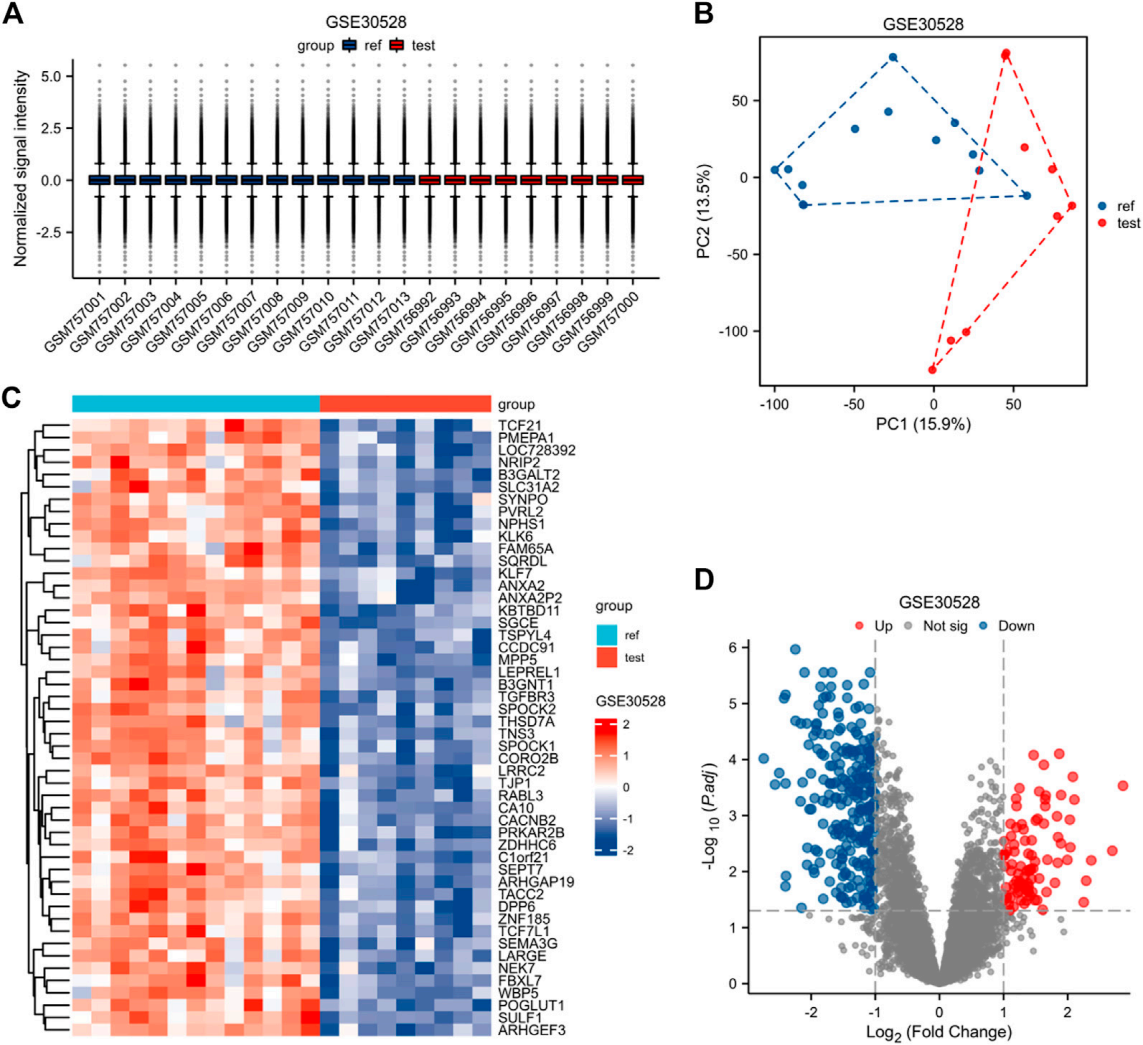
Normalized expression matrices **(A)** of the GSE30528 dataset. Differentially expressed genes from the GSE30528 dataset with a |log2 FC| 1 screening criterion and an adjusted *p*-value of 0.05 **(B–D)**. [**(B)** PCA plot; **(C)** Heatmap plot; and **(D)** Volcano plots of GSE30528. PCA: Principal component analysis; Ref: Control group; Test: Diabetic nephropathy, DN].

**FIGURE 4:**
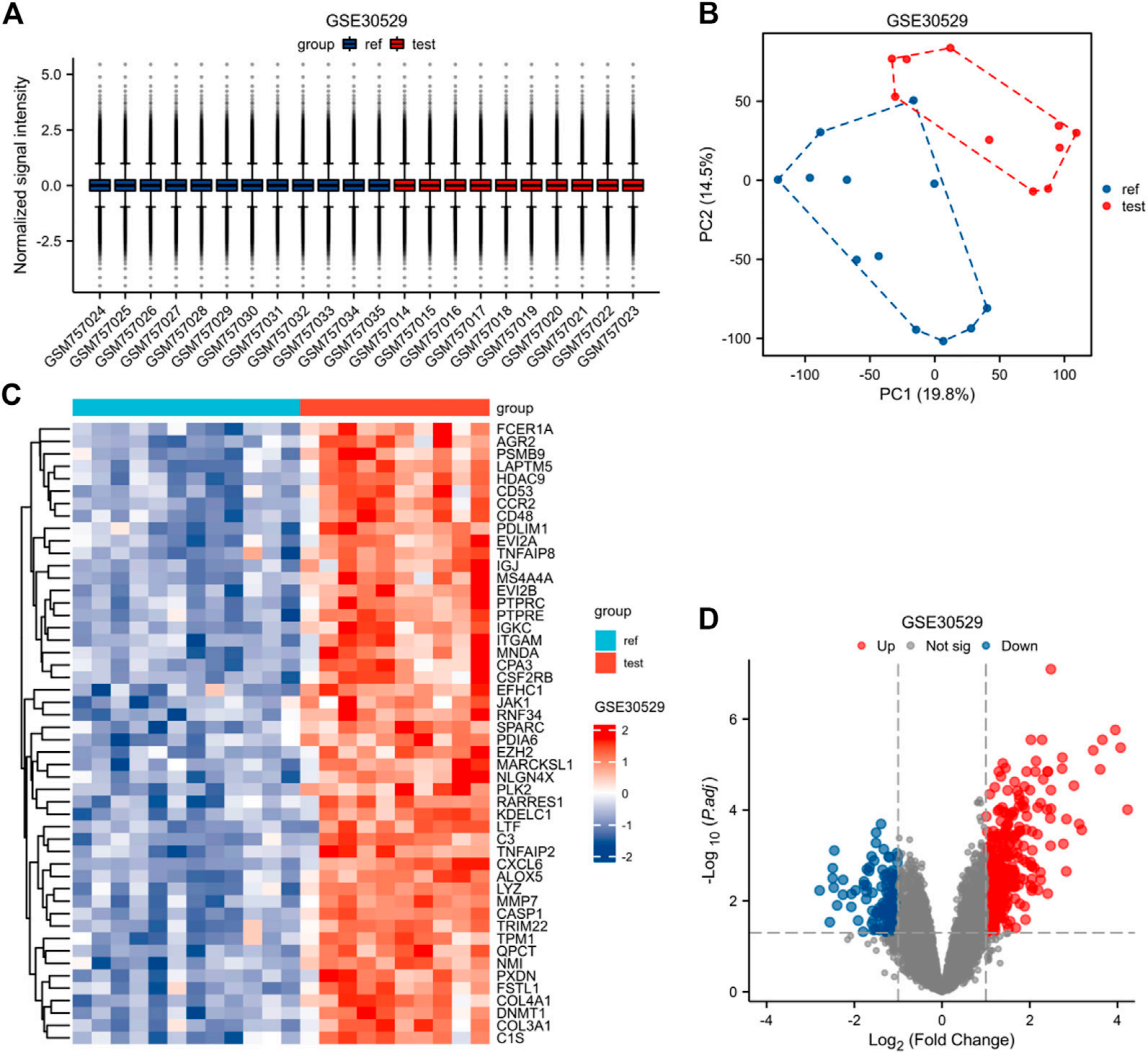
Normalized expression matrices **(A)** of the GSE30528 dataset. Differentially expressed genes of the GSE30528 dataset with a filtering standard of |log2 FC|≥1 and adjust *p*-value < 0.05**(B–D)**. [**(B)** PCA plot; **(C)** Heatmap plot; **(D)** Volcano plots of GSE30528. PCA: Principal Component Analysis; Ref: Control Group; Test: Diabetic Nephropathy, DN).

The expression matrices of GSE30122, GSE30528, and GSE30529 for the three data sets were normalized, and the box plots’ distribution tendency was generally straight (Figures 2A–4A). The probes associated with 12,548 genes in the GSE30122 dataset were identified, and the DEGs for DN were confirmed. |log2(FC)|>1 and p. adj0.05 were met by 120 IDs. Under this threshold, the number of high expressions in the DN group was 86 and in the reference group was 34. Normalization is performed through the inter-array normalization function of the Limma package and then visualized. The GSE30528 (Figure 3) and GSE30529 (Figure 4) datasets were analyzed based on the same criteria and visualized as normalized box plots, volcano plots, heatmaps, and PAC plots.

### GO enrichment analysis

**FIGURE 5:**
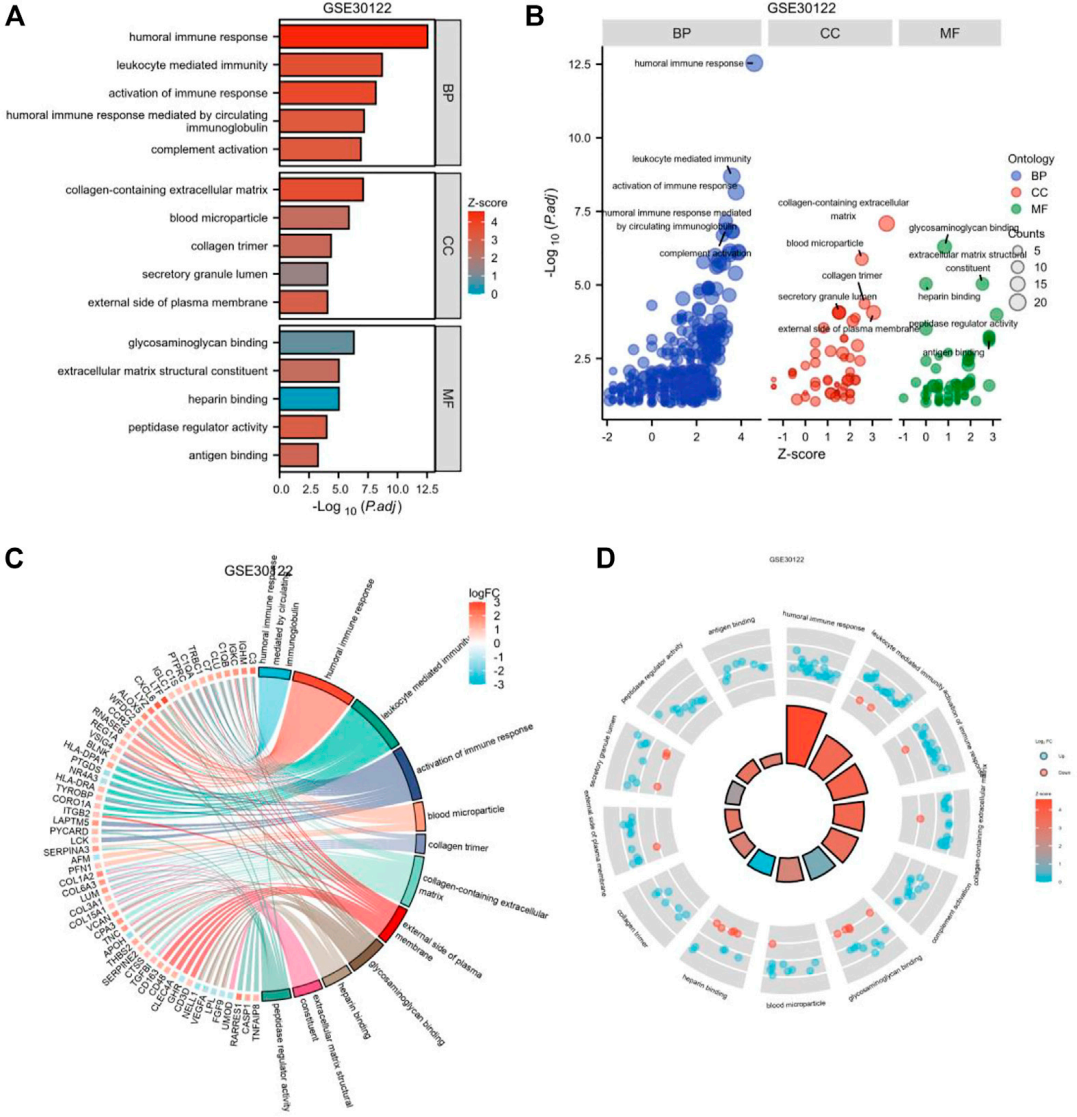
Enrichment plots through GO. [**(A)** Bar graph of GO enrichment pathways, (**B)** Bubble plot, **(C)** chord diagram, **(D)** loop graph.].

To further investigate the biofunction of the 120 DEGs obtained in GSE 30122, GO functional enrichment analysis was performed. There were 304 items for BP, 57 items for CC, and 52 items for MF based on the adjusted filtering criteria (*p*-value < 0.05 and Q value < 0.2). GO analysis of the enrichment showed that the differentially expressed genes were mainly functional in the following 15 ways (Table 2): GO: 0006959, “humoral immune response.” GO: 0002253, “activation of the immune response.” GO: 0002443, “leukocyte-mediated immunity.” ( *www.spandidos-publications.com* ) GO: 0006956, “complement activation.” GO:0002455-humoral immune response mediated by circulating immunoglobulin (*draco.cyverse.org*); GO:0062023-collagen-containing extracellular matrix; GO: 0072562 (blood microparticle); GO: 005581 (collagen trimer); GO: 0034774 (secretory granule lumen); GO: 009897 (external side of plasma membrane); GO: 0005539—Glycosaminoglycan binding; GO: 0008201—Heparin binding; GO: 1901681—Sulfur compound binding; GO: 0003823—Antigen binding; GO: 0005201—Extracellular matrix structural constituent ( *www.ncbi.nlm.nih.go* ). The results are presented in Table 2. In order to sufficiently demonstrate the requirements of GO enrichment analysis, the R packages GOplot and ggplot2 were employed for visualization. (Figure 5).

**TABLE 2:** GO terms and pathways significantly enriched by DEGs.

ONTOLOGY	ID	Description	GeneRatio	BgRatio	pvalue	p.adjust	qvalue
BP	GO:0006959	humoral immune response	22/110	317/18800	9.46E-18	2.30E-14	1.74E-14
BP	GO:0002253	activation of immune response	19/110	386/18800	9.75E-13	1.18E-09	8.95E-10
BP	GO:0002443	leukocyte mediated immunity	20/110	457/18800	2.01E-12	1.63E-09	1.23E-09
BP	GO:0006956	complement activation	12/110	131/18800	1.55E-11	9.39E-09	7.11E-09
BP	GO:0002455	humoral immune response mediated by circulating immunoglobulin	11/110	121/18800	1.23E-10	5.99E-08	4.53E-08
CC	GO:0062023	collagen-containing extracellular matrix	18/114	429/19594	5.47E-11	1.10E-08	8.18E-09
CC	GO:0072562	blood microparticle	11/114	147/19594	9.65E-10	9.75E-08	7.21E-08
CC	GO:0005581	collagen trimer	8/114	86/19594	3.73E-08	2.51E-06	1.86E-06
CC	GO:0009897	external side of plasma membrance	13/114	455/19594	2.48E-06	9.52E-05	7.04E-05
CC	GO:0034774	secretory granule lumen	11/114	322/19594	2.84E-06	9.52E-05	7.04E-05
MF	GO:0005539	glycosaminoglycan binding	13/112	234/18410	1.81E-09	5.63E-07	4.10E-07
MF	GO:0008201	heparin binding	10/112	168/18410	7.78E-08	1.01E-05	7.32E-06
MF	GO:0005201	extracellular matrix structural	10/112	172/18410	9.71E-08	1.01E-05	7.32E-06
MF	GO:0003823	antigen binding	9/112	174/18410	1.17E-06	8.82E-05	6.41E-05
MF	GO:0061134	peptidase regulator activity	10/112	230/18410	1.42E-06	8.82E-05	6.41E-05

*GO, gene ontology; DEGs, Differentially Expressed Genes; MF, molecular fuction; CC, cellular component; BP, biological process.

### KEGG pathways enrichment analysis

**FIGURE 6:**
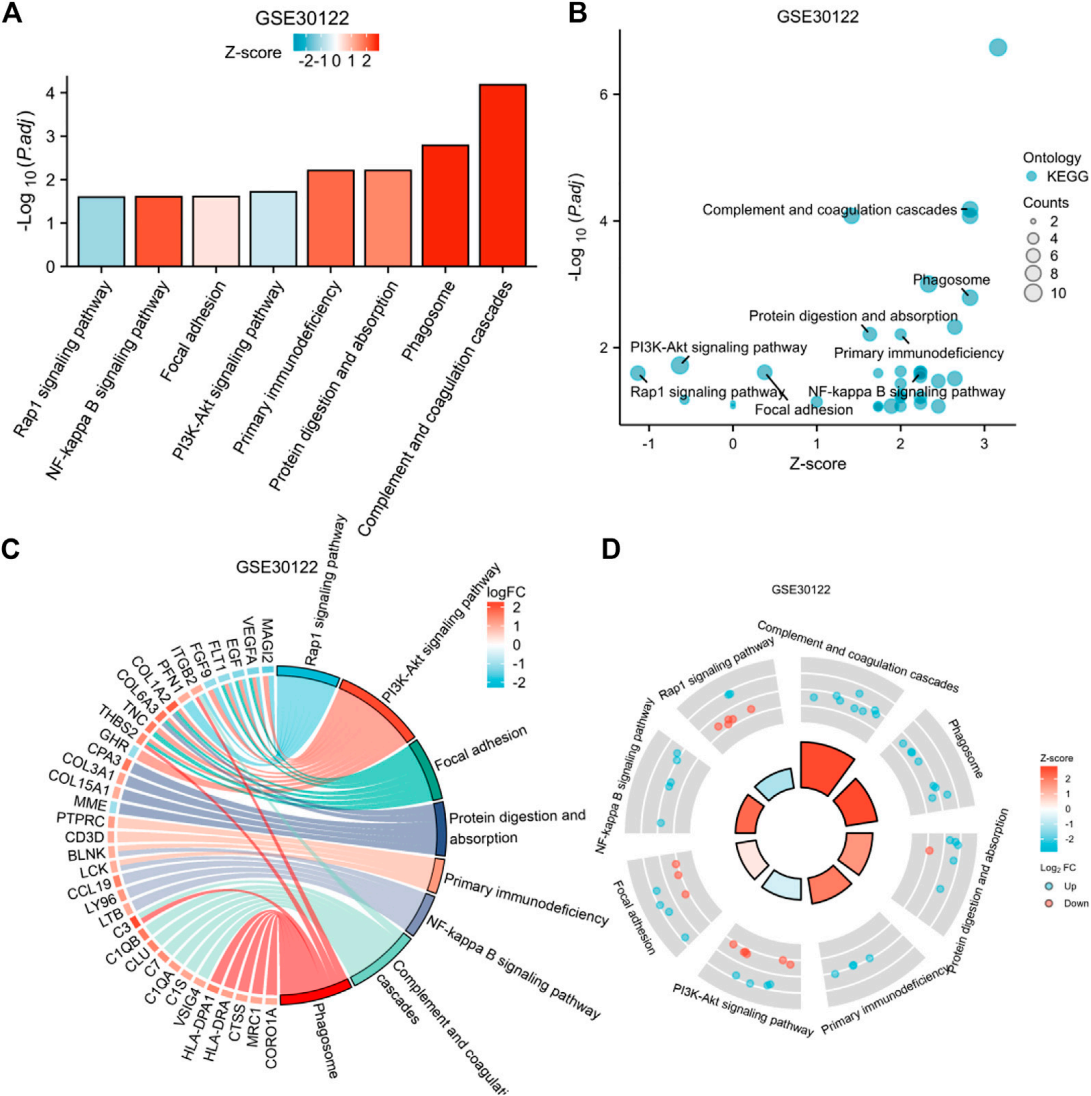
Enrichment plots through KEGG. [**(A)** Bar graph of KEGG enrichment pathways, **(B)** Bubble plot, **(C)** chord diagram, **(D)** loop graph).

To explore the potential biological pathways in diabetic nephropathy, we applied DEGs for KEGG pathway analysis. We used DEGs for KEGG pathway analysis after adjusting the filtering criteria (P and Q values). The adjusted filtering criteria (*p*-value 0.05 and Q-value 0.2) indicate that 21 KEGG pathways were enriched in GSE 30122. Complement and coagulation cascades; The phagosome; protein digestion and absorption; the PI3K-Akt signaling pathway; Primary immunodeficiency; Focal adhesion; The NF-kappa B signaling pathway; The Rap1 signaling pathway; and other pathways were enriched (Figure 6). Based on these findings, pathways such as inflammation, the immune response, and mediated interstitial renal fibrosis may be involved in the biological pathways of diabetic nephropathy.

### GSEA analysis of enrichment

**FIGURE 7:**
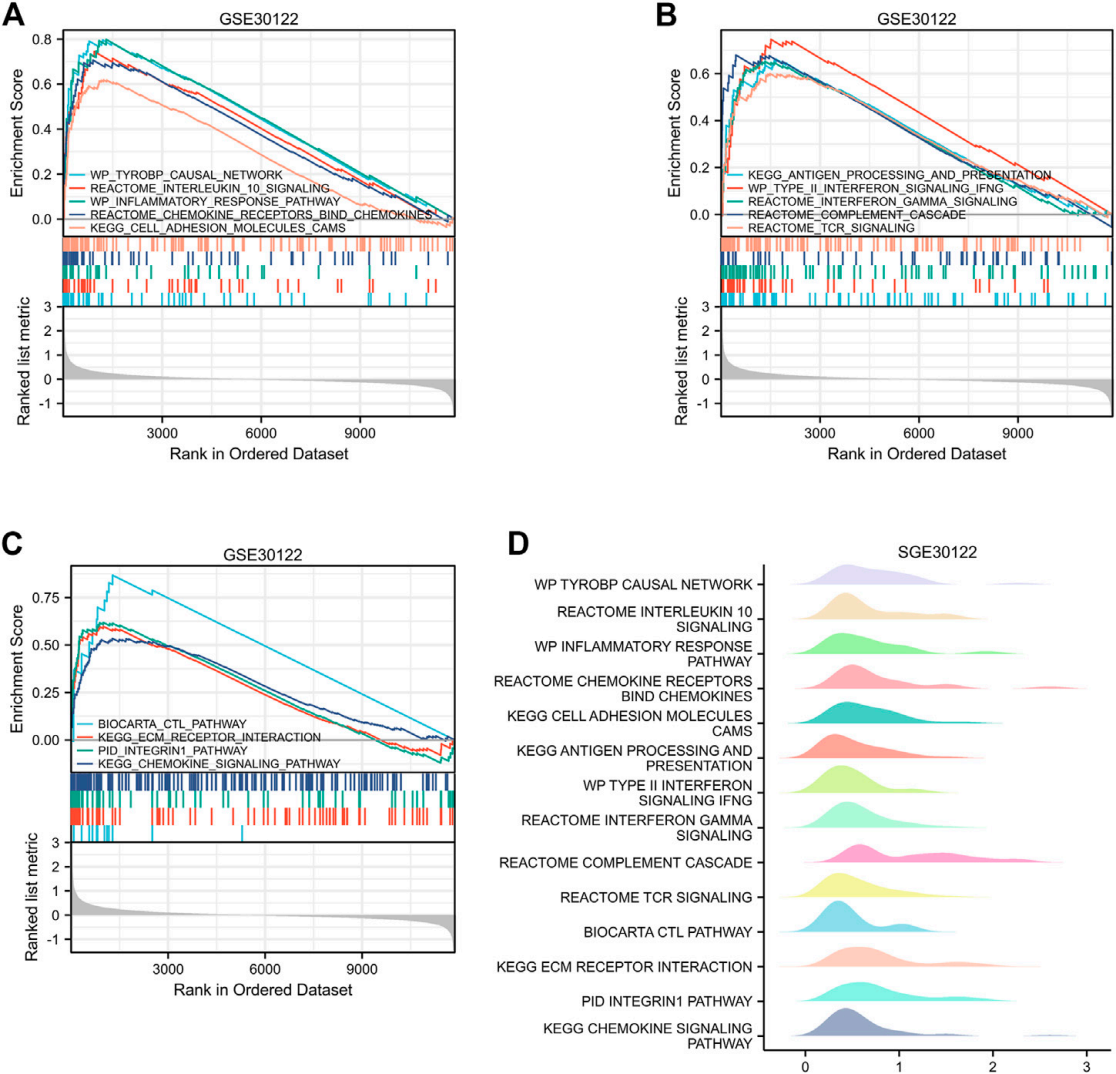
Enrichment plots by GSEA. [**(A–C)** GSEA visual analysis, **(D)** GSEA ridgeplot).

The GEO30122 dataset was examined using GSEA to characterize the functional genome associated with diabetic nephropathy. Finally, a total of 226 datasets met the FDR Q value <0.25 and the p. adjust value <0.05. We selected 14 of these pathways that met NES ≥ 2.0 and the adjusted *p*-value < 0.05 for GSEA enrichment analysis to be shown (Table 3). These data sets include (Cho et al., 2018): TYROBP causal network (GBD Chronic Kidney Disease Collaboration, 2020); Interleukin 10 signaling (Wang et al., 2021); Inflammatory response pathway (Liu et al., 2021); Involved in chemokine receptor-binding (Ma et al., 2019); Involved in cell adhesion molecules, CAMS (Bakris and Molitch, 2014); Interferon γand α signaling pathways (Macisaac et al., 2014); Type II interferon signaling, IFNG (Sourris and Forbes, 2009); Antigen processing and presentation (Persson and Rossing, 2018); Reactome complement cascade (Bjornstad et al., 2015); Reactome tcr signaling (MacIsaac and Jerums, 2011); Biocarta CTL pathway (MacIsaac and Ekinci, 2019); ECM receptor interaction (Zhou et al., 2021); PID integrin1 pathway (MacIsaac et al., 2015); Chemokine pathway; and so on. Then, the results of the GSEA enrichment analysis were visualized and presented (Figure 7).

**TABLE 3:** Analysis of GSEA enrichment.

ID/Description	SetSize	EnrichmentScore	NES	pvalue	p.adjust	qvalues	rank	leading_edge
WP_TYROBP_CAUSAL_NETWORK	50	0.795981415	2.503	0.0017	0.03269	0.02680	1194	tags = 54%, list = 10%, signal = 49%
REACTOME_INTERLEUKIN_10_SIGNALING	43	0.744766999	2.281	0.0017	0.03269	0.02680	937	tags = 40%, list = 8%, signal = 37%
WP_INFLAMMATORY_RESPONSE_PATHWAY	29	0.798773879	2.237	0.0019	0.03269	0.02680	1299	tags = 52%, list = 11%, signal = 46%
REACTOME_CHEMOKINE_RECEPTORS_BIND_CHEMOKINES	52	0.706657097	2.232	0.0017	0.03269	0.02680	900	tags = 37%, list = 8%, signal = 34%
KEGG_CELL_ADHESION_MOLECULES_CAMS	115	0.618803129	2.209	0.0016	0.03269	0.02680	1223	tags = 35%, list = 10%, signal = 32%
KEGG_ANTIGEN_PROCESSING_AND_PRESENTATION	73	0.661032843	2.201	0.0016	0.03269	0.02680	1669	tags = 42%, list = 14%, signal = 37%
WP_TYPE_II_INTERFERON_SIGNALING_IFNG	36	0.74575286	2.193	0.0018	0.03269	0.02680	1512	tags = 58%, list = 13%, signal = 51%
REACTOME_INTERFERON_GAMMA_SIGNALING	77	0.652216095	2.184	0.0016	0.03269	0.02680	1333	tags = 42%, list = 11%, signal = 37%
REACTOME_COMPLEMENT_CASCADE	54	0.679372563	2.155	0.0017	0.03269	0.02680	447	tags = 24%, list = 4%, signal = 23%
REACTOME_TCR_SIGNALING	106	0.600536288	2.113	0.0016	0.03269	0.02680	1481	tags = 33%, list = 12%, signal = 29%
BIOCARTA_CTL_PATHWAY	13	0.867303264	2.028	0.0019	0.03269	0.02680	1283	tags = 85%, list = 11%, signal = 76%
KEGG_ECM_RECEPTOR_INTERACTION	79	0.598503792	2.017	0.0016	0.03269	0.02680	994	tags = 30%, list = 8%, signal = 28%
PID_INTEGRIN1_PATHWAY	62	0.61879348	2.015	0.0017	0.03269	0.02680	994	tags = 39%, list = 8%, signal = 36%
KEGG_CHEMOKINE_SIGNALING_PATHWAY	165	0.534914996	2.003	0.0015	0.03269	0.02680	1276	tags = 24%, list = 11%, signal = 22%

*NES, normalized enrichment score.

### Construction of PPI network and screening of hub genes

**FIGURE 8:**
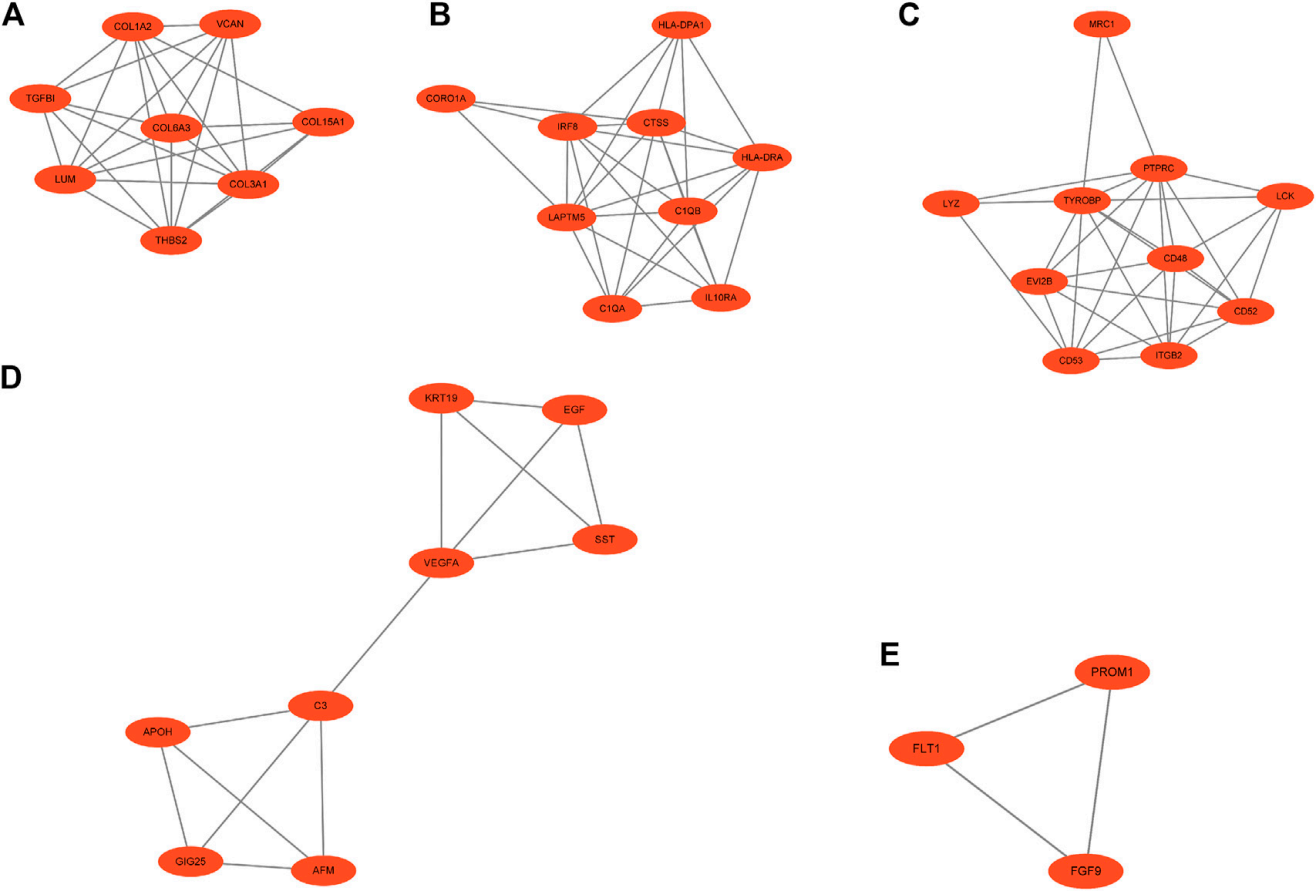
PPI network construction in GSE30122. [**(A–E)** Sub-network diagram constructed by the MCODE plugin].

**FIGURE 9:**
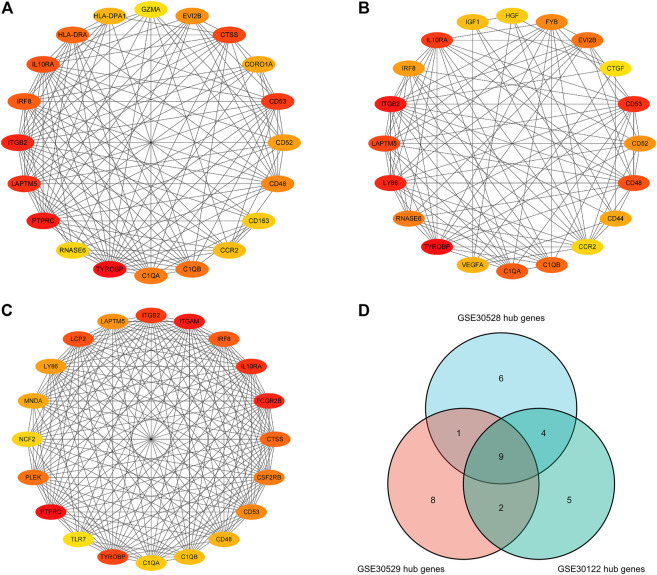
Hub genes in the GSE30122, GSE30528 and GSE30529 datasets. **(A)** Top 20 hub genes in GSE30122; **(B)** Top 20 hub genes in GSE39528; **(C)** Top 20 hub genes inGSE30529 dataset; **(D)** Common hub genetic Venn diagram.

Separate PPI analyses were conducted for each of the three datasets using the STRING platform. The final 115 nodes and 448 interactions were identified in the GSE30122 dataset. 338 nodes and 973 edges were identified in dataset GSE30528. A total of 457 nodes and 2946 edges were identified in dataset GSE30529. A sub-network graph was constructed by the MCODE plugin for the differential genes of the GSE30122 dataset (Figure 8). In addition, the MCC module in the Cytohubba plugin filtered the top 20 hub genes in each of the three datasets and then took the intersection of the top 20 hub genes to determine the common hub genes by Venn software, and finally a total of 9 common hub genes were obtained (TYROBP, ITGB2, CD53, IL10RA, LAPTM5, CD48, C1QA, C1QB, and IRF8) (Figure 9).

### mRNA-miRNA and mRNA-TF regulatory network

**FIGURE 10:**
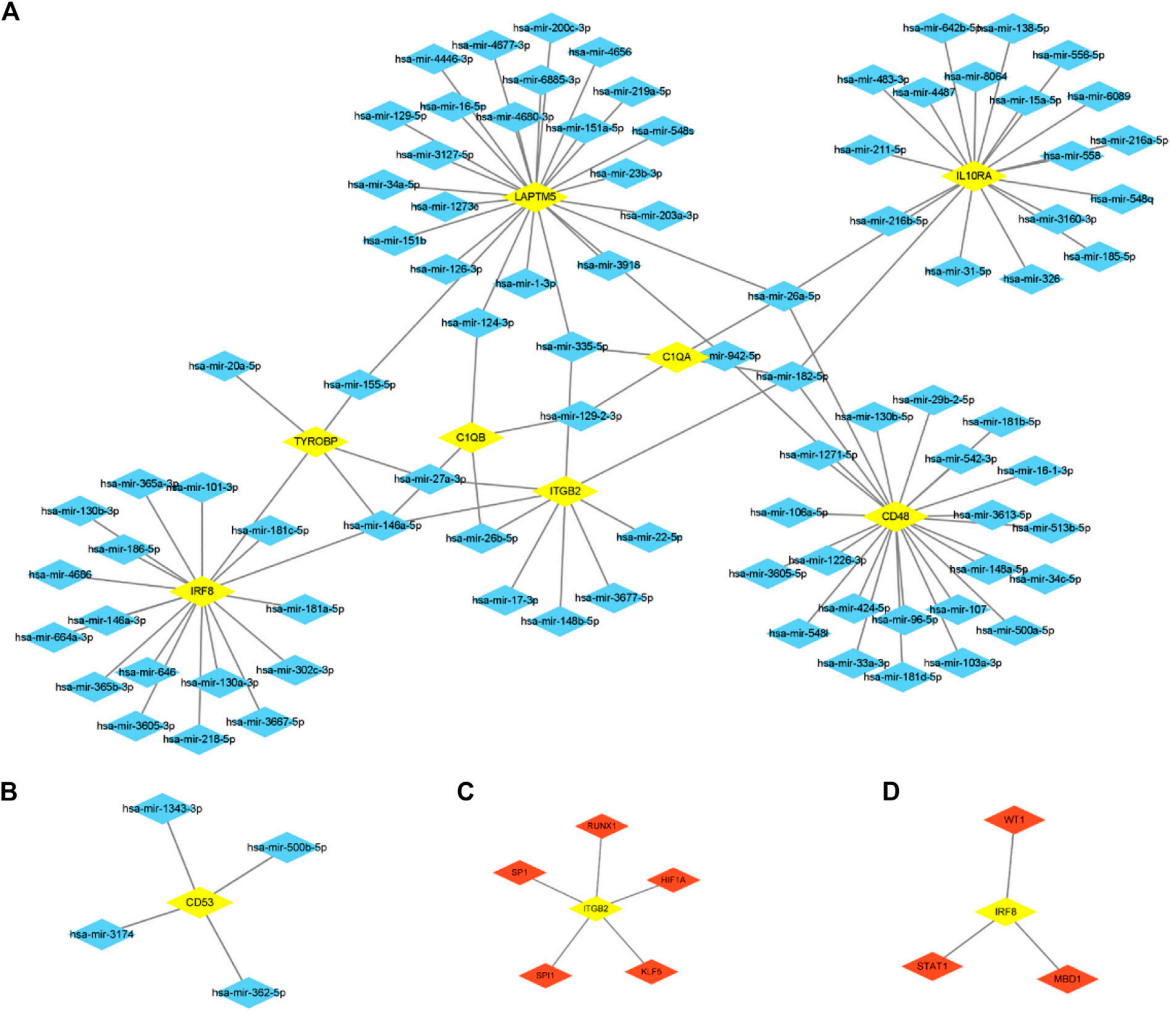
mRNA-miRNA regulatory network **(A, B)** and MRNA-TF regulatory network **(C, D)**.

We predict target miRNAs and TFs using the miRNet tool. Lastly, we identified 93 miRNAs from nine common hub genes and identified 110 mRNA-miRNA pairs. Meanwhile, we identified 8 TFs for 2 common hub genes and identified 8 mRNA-TF pairs. Based on the forecast results, a co-expression network of mRNAs and miRNAs consisting of 93 nodes and 110 edges and an expression network graph of mRNAs and TFs consisting of 10 nodes and 8 edges were constructed using Cytoscape. (Figure 10). With 18 miRNAs modulating IRF8, 4 miRNAs modulating TYROBP, 4 miRNAs modulating C1QB, 9 miRNAs modulating ITGB2, 4 miRNAs modulating C1QA, 24 miRNAs modulating LAPTM5, 24 miRNAs modulating CD48, 19 miRNAs modulating IL10RA, and 4 miRNAs modulating CD53.

### Identification of potential key genes for upstream diseases associated with diabetic nephropathy

**FIGURE 11:**
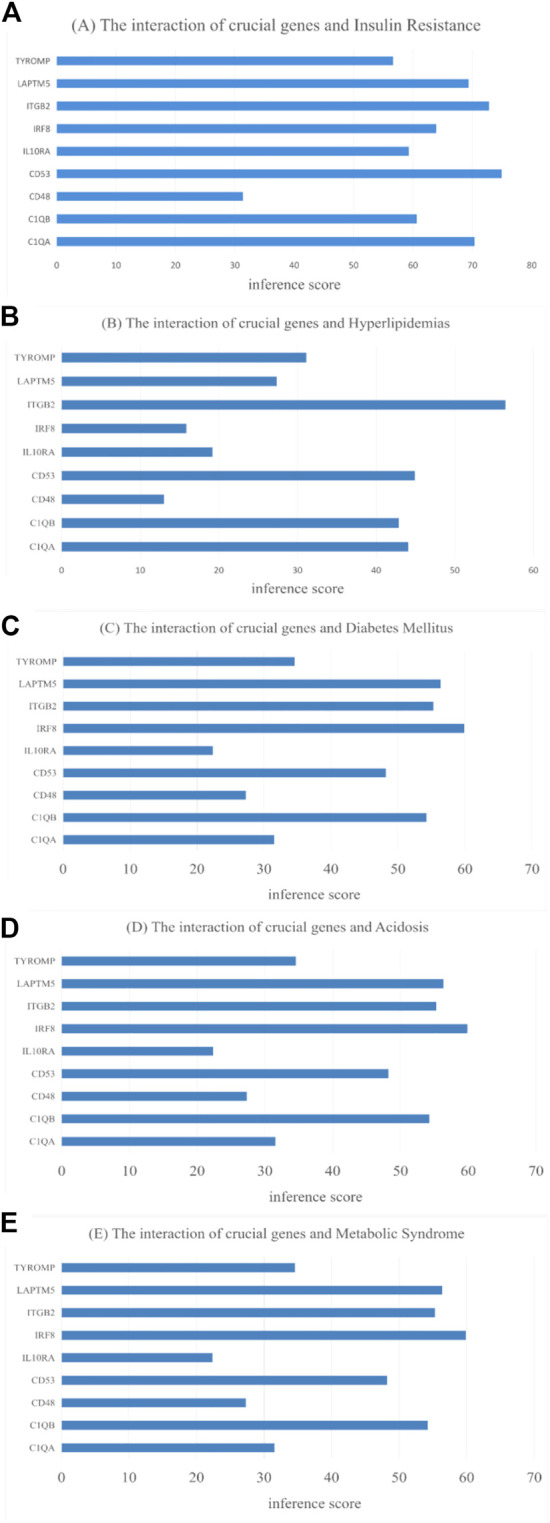
CTD analysis of the association between potential key genes and disease. **(A)** Insulin Resistance; **(B)** Hyperlipidemias; **(C)** Diabetes Mellitus; **(D)** Acidosis; **(E)** Metabolic Syndrome.

Use of CTD to probe the interactions of potential key genes with diseases associated with diabetic nephropathy As shown in Figure 11, there are potential key genes for insulin resistance, hyperlipidemias, diabetes mellitus, acidosis, and the metabolic syndrome. The inferred scores in the CTD reflect associations between chemicals, diseases, and genes. The results of the interactions show that LAPTM5, IRF8, IGTB2, CD53, and C1QB scored higher with diabetes mellitus.

### GSE30528 and GSE30529 validate the expression and diagnostic value of nine common hub genes

**FIGURE 12:**
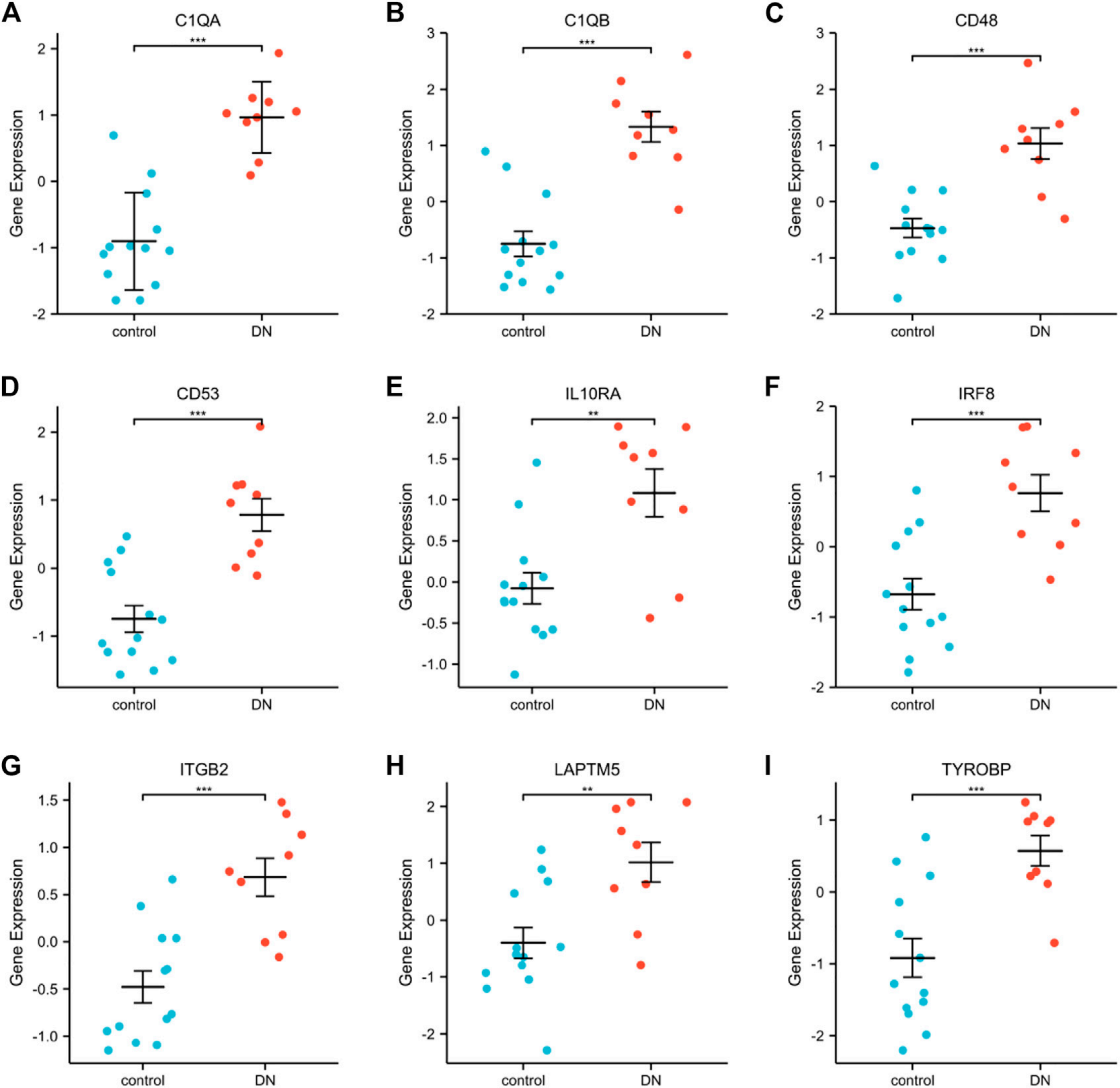
Expression comparison of nine DN-related hub genes in the GSE30528 dataset **(A–I)**. (**, *p* < 0.01; ***, *p* < 0.001).

**FIGURE 13:**
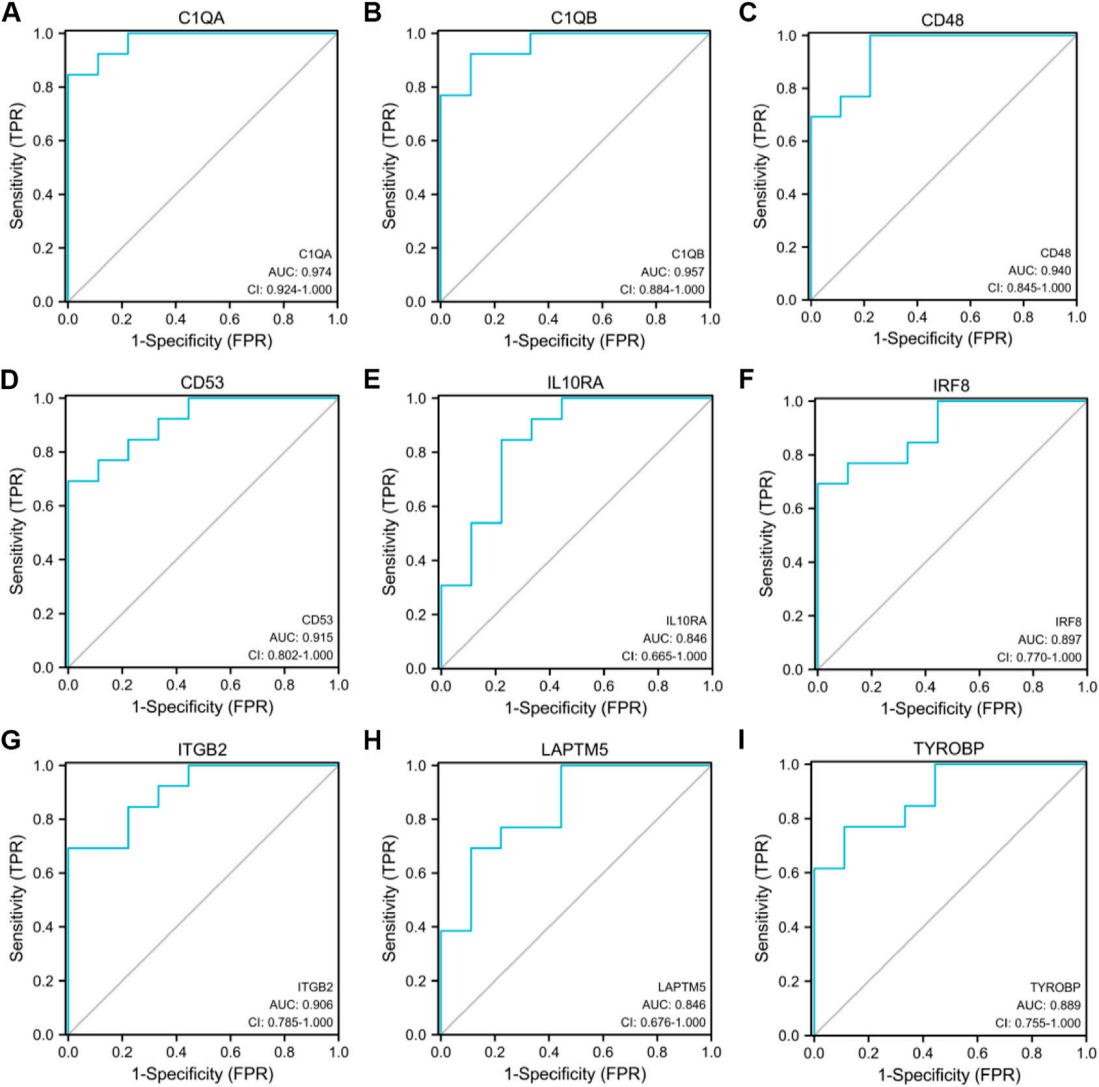
Diagnostic ROC curves for 9 common hub genes associated with DN in the GSE30528 dataset **(A–I)**. (ROC, receiver operating characteristic; TPR, true positive rate; FPR, false positive rate).

**FIGURE 14:**
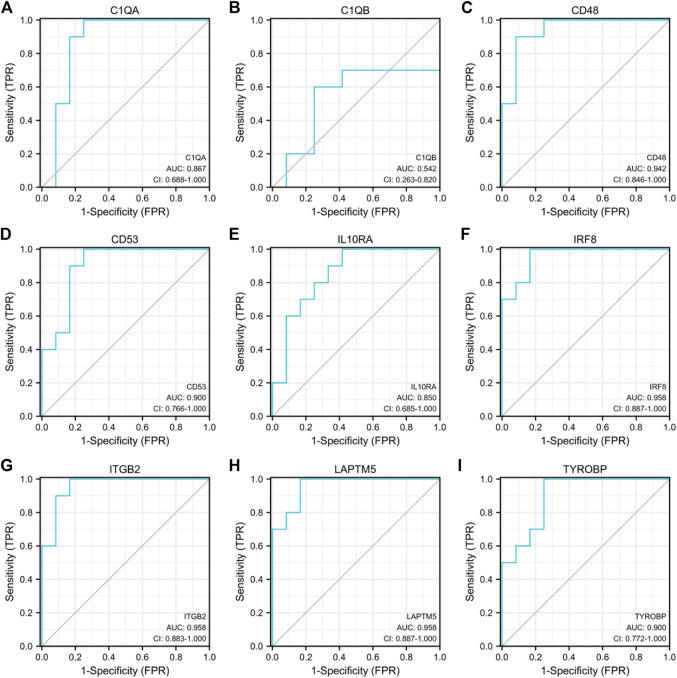
Diagnostic ROC curves for 9 common hub genes associated with DN in the GSE30529 dataset **(A–I)**.

**FIGURE 15:**
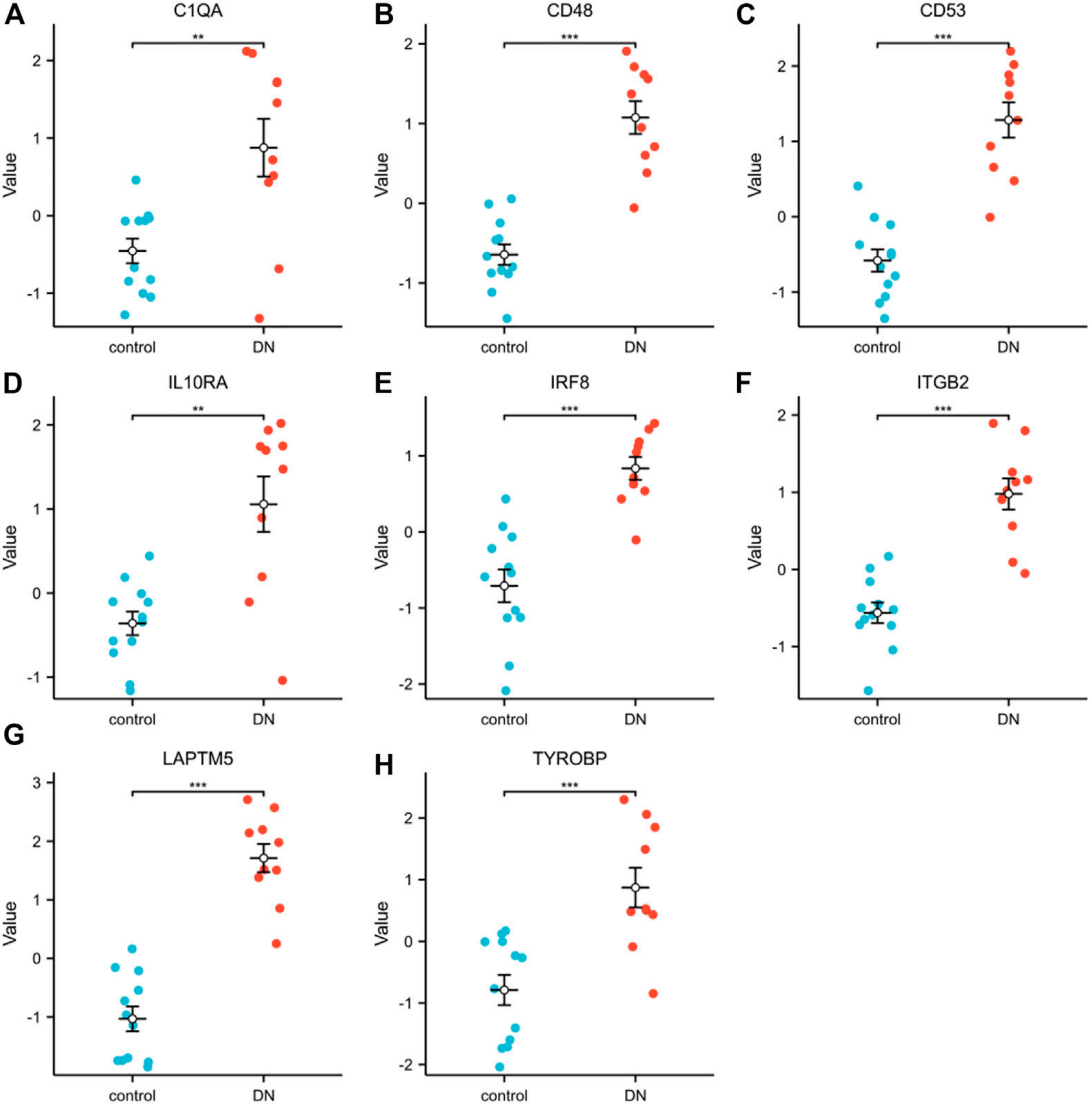
Expression comparison of eight DN-related hub genes in the GSE30529 dataset. (**, *p* < 0.01; ***, *p* < 0.001)..

GSE30528 was used to detect the expression of the screened common hub genes, and the expression of 9 diagnostically relevant hub genes (TYROBP, ITGB2, CD53, IL10RA, LAPTM5, CD48, C1QA, C1QB, and IRF8) differed between DN and normal control patients (Figures 12A–I). We created ROC curves using data from patients with diabetic nephropathy compared to healthy individuals. Findings suggest that these eight genes have important value in the diagnosis of diabetic nephropathy. In the GSE30528 dataset, nine common hub genes all had a good diagnostic value for DN (Figure13A–I). In the GSE 30529 dataset, the genes C1QA, CD48, CD53, IL10RA, IRF8, ITGB2, LAPTM5, and TYROBP all had good diagnostic values; however, the AUC of the variable C1QB was 0.542 (95% CI 0.263–0.820), which was not diagnostic (Figure14A–I). Eight Hub genes' expression in the normal group and DN group in the GSE30529 dataset had also been analyzed and visualized (Figures 15A–H).

## Discussion

Diabetic nephropathy (DN) is the major cause of CKD and ESRD (Vaisar et al., 2018), and it has become a global public problem. In recent years, DN has achieved significant progress in the diagnosis, treatment, and prevention of the disease, and a wealth of research results have been obtained. SGLT2 inhibitors and GLP1 receptor agonists have shown a significant advance in renal protection. Inhibition of apoptosis signal-regulated kinase 1 (ASK1) by histone modifications in sufficient cells induces oxidative stress to reduce glomerular injury. Bioinformatics research on biomarkers has also achieved significant advances and gained increasing attention. Studying biomarkers of diabetic nephropathy is particularly important for early diagnosis, therapy, and evaluation of the disease’s prognosis. For this research, we utilized 19 DN samples and 50 gene expression profiles of normal subjects included in the GSE30122 dataset, and the data were analyzed using biological informatics tools. 120 IDs met the thresholds of |log2(FC)| ≥ 1 and p. adj < 0.05. Within this threshold, 86 IDs were hyper-expressed in the DN groups and 34 IDs were hyper-expressed in the control groups for GO, KEGG, and GSEA analysis. Top20 hub genes were filtered with the Cytohubba plugin, and 9 common hub genes were obtained by taking the intersection with the Top20 gene Venn diagram of the GSE30528 and GSE30529 datasets. Further validation of the diagnostic value was performed using ROC curves in the GSE30528 and GSE30529 datasets, and finally, some important hub genes such as TYROBP, ITGB2, CD53, IL10RA, LAPTM5, CD48, C1QA, and IRF8 were associated with a risk for DN, suggesting that these may play an important role in the mechanisms of DN onset and progression.

There is tremendous heterogeneity in DN susceptibility, and genetic regulation may be an essential factor contributing to this heterogeneity. The mainly damaged cells in DN are podocytes, and the severity of damage is highly related to disease progression. Podocyte apoptosis is the leading cause of podocyte reduction in DN (Li et al., 2007). ROS-mediated apoptosis of podocytes induced by hyperglycemia is the initial step in the progression of diabetic nephropathy (Stern et al., 2016). Macrophage infiltration is an important distinguishing feature of DN (Tesch, 2010). Li et al. (2022a) reported that the differential gene expression, signaling pathways involved, and signature enrichment profiles obtained differed significantly by the proportion of cell types in different datasets. By integrated transcriptome analysis, two genes (TEKT2 and PIAS2) related to spermatogenesis were found to be dysregulated to mediate DN, and the knockdown of TEKT2 could resist high glucose induction of podocyte cytoskeletal remodeling and NPHS1 protein downregulation. In our study, C1Q8 was found to be highly expressed in both DN glomeruli and tubular cells, but its diagnostic value in DN tubular cells was less (AUC = 0.542).

TYROBP is located on chromosome 19 and can act as an adaptor molecule for TREM (trigger receptor expressed on myeloid cells) and induce cytokine production in macrophages (Colonna, 2003), and is involved in the regulation of interleukin-1β, interleukin-6, and interleukin-10. TYROBP was identified by bioinformatics analysis as a potential candidate gene for lupus nephritis (Zhang et al., 2020) and a candidate gene for tubulointerstitial fibrosis in diabetic nephropathy, possibly associated with the epithelial-mesenchymal transition of the renal tubular epithelium (Bai et al., 2023). ITGB2 is a protein-encoding gene involved in processes such as apoptosis, cell adhesion, cell-matrix adhesion, and inflammatory responses. The encoded protein can be linked to endothelial cell surface adhesion molecules and various cytoskeletal proteins and is involved in signal transduction, possibly accelerating small vessel lesions in DN *via* the cell adhesion molecule (CAM) pathway. (Geng et al., 2019). Ligand adhesion molecules are part of the immunoglobulin family, are highly expressed in the serum and kidney of DN, and can contribute to disturbed lipid metabolism in podocytes (Fu et al., 2020). CD53 is a critical factor in the regulation of immune cells and is found in cellular exosomes (Buschow et al., 2010), cell surfaces, and plasma membranes. It is involved in signal transduction and may contribute to inflammation and apoptosis in DN through immune cell infiltration and exosome secretion. (Jiang et al., 2022). IL10RA, the interleukin 10 receptor subunit alpha, is involved in the negative regulation of autophagy, the positive regulation of the JAK-STAT receptor signaling pathway, and the response to lipopolysaccharide. The JAK-STAT pathway has an essential effect on the progression of DN by promoting inflammatory factor expression and inducing the activation of inflammatory cells (Zhang et al., 2017). Lysosomal exhaustion leads to dysfunctional autophagy in kidney tubular epithelial cells, and SMAD3, a key effector of TGFB-SMAD signaling, causes tubular epithelial damage in diabetic organisms by disrupting the autophagic flow, which in turn accelerates the DN process (Yang et al., 2021). LAPTM5 encodes a lysosome-associated transmembrane receptor that is involved in the induction of programmed cell death (Inoue et al., 2009), the positive regulation of NIK/NF-kappaB signaling (*rgd.mcw.edu*), and the positive regulation of the MAPK cascade (Adra et al., 1996). Studies suggest that NF-kappaB receptor activation may contribute to podocyte injury in combination with cytokines such as TNF, MAC-2, and IL-1B, promoting glomerular oxidative stress and pro-inflammatory factor production and mediating the development of DN (38). CD48, which encodes immunoglobulin-like receptors, is involved in defense responses. Diabetic nephropathy is a multi-mechanism disease involving genetic, inflammatory, immune, and endocrine mechanisms. Autoantibodies produced by B cells can lead to the deposition of immune complexes in the kidney (Sosenko et al., 2017), inducing the aggregation of macrophages, generating a cascade response, and aggravating the progression of diabetic nephropathy. C1QA, the complement C1q A chain, is involved in complement activation. Studies have suggested that complement activation may be a major cause of DN (Ricklin et al., 2018). C1QA and ITGB2 are involved in the complement cascade response, CD48 and CD53 may be involved in humoral immunity and macrophage activation, and integrated polygenic regulation promotes the inflammatory response cascade effect and accelerates DN progression (Klessens et al., 2017; Xu et al., 2021). IRF8, which is highly expressed in the DN group, is involved in autophagy, immune response, phagocytosis, and regulation of interferon production. IRF8 is an important regulatory gene for the development of dendritic cells, which play a crucial role in the regulation of insulin secretion and hyperglycemia (Besin et al., 2011). High glucose promotes dendritic cell maturation through activation of the NF-kB pathway, accelerating and amplifying the inflammatory immune response and accelerating the development of DN (Tu et al., 2019). The identification of these molecular biomarkers might be used for diagnosis, therapy, and prediction of diseases, and the regulation of disease regression from molecular mechanisms might be an important tool for future individualized treatment.

The mechanism of DN is very sophisticated, and present treatments can only slow down its development but cannot effectively prevent and cure it. The pathophysiology of DN is often believed to include problems in hemodynamics, metabolic function, and hormone production. Advanced glycosylation end products (AGE), renin-angiotensin-aldosterone system (RAAS), transforming growth factor-1 (TGF-1) expression, activation of protein kinase C (PKC), mitogen-activated protein kinase (MAPK), and reactive oxygen species (ROS) are all considered to be significant pathways in the initial stages and progression of diabetic nephropathy. However, various pathway factors regulate each other and overlap (Samsu, 2021). Functional enrichment analysis revealed that the differential genes might be engaged in biologic processes as immune response, antigen-antibody activation, and complement activation, promoting the development of DN through phagocytosis vesicles, the PI3K-Akt signaling pathway, focal adhesion, the NIK/NF-kappaB signaling pathway, and the Rap1 signaling pathway. The pathological mechanisms associated with the participation of ECM in DN development have potential interactions with immune cells. Li et al. (2022b) showed that the hub genes of DN patients are mainly enriched in those involved in ECM-receptor interactions, focal adhesion, complement, and coagulation cascade reactions, a result that is consistent with our findings. They also inferred that COL6A3, COL1A2, THBS2, CD44, and FN1 promote the progression of DN through the ECM-receptor interaction pathway and are expected to be new therapeutic targets. A variety of inflammatory factors and tissue factors are the major inducers and drivers of renal inflammation and plays a major part in the network of pro-inflammatory molecules in the DN. In patients with DN, the PI3K-Akt pathway has been demonstrated to be an important signaling pathway (Chen et al., 2013). Lu et al. (2022) found that LCK and HCK genes were highly expressed in DN through bioinformatics analysis of the role of immune-related genes in DN progression and identified two different immune-related subgroups, C1 and C2, which provided a theoretical basis for the formulation of immunotherapy for DN patients. In addition, enrichment analysis indicated that adhesion molecules may be involved in the integrin pathway closely related to DN, similar to previous reports (Gu et al., 2012). According to the analysis of published literature, C1S and C1R are differentially expressed in DN (Zhang et al., 2009), suggesting that C1 may be involved in the development of DN. Another clinical study showed a sixfold increase in glomerular C3 levels in renal biopsy samples from patients with DN (Woroniecka et al., 2011), which suggests that the complement system may have a positive role in DN and glomerulosclerosis.

In our study, Transcription factors HIF1A, KLF5, RUNX1, SP1, SPI1, STAT1, MBD1 and WT1 may be related to diabetic nephropathy. The results were similar to previous studies (Hu et al., 2021). Chronic hyperglycemia can lead to microcirculatory disorders presenting with renal ischemia and hypoxia, and hypoxia can lead to inhibition of HIF-1α stability and function and decreased renal hypoxia tolerance. Studies have shown that HIF-1 is repressed in DN renal tubules (Gu et al., 2013), and tubular hypoxia promotes tubular atrophy and interstitial fibrosis, facilitating the progression of glomerular lesions in DN. High expression of HIF-1 in DN in mesangial cells accelerates the process of renal fibrosis (Isoe et al., 2010). Sp1 mediates the upregulation of Prdx6 expression to prevent diabetic nephropathy by alleviating oxidative stress and ferritin deposition, thereby preventing podocyte damage (Zhang et al., 2021a). Animal experiments showed that inhibition of KLF5 expression alleviated foot cell injury in diabetic neuropathy (Zhang et al., 2021b), Runx1 promoted TGFβ1-induced kidney fibrosis in mice by upregulating the PI3K pathway (Zhang et al., 2021c), STAT1 phosphorylation inhibited the M1 phenotypic transformation of macrophages and suppressed DN progression (Zhang et al., 2019), and WT1-induced apoptosis in diabetic nephropathy podocytes by activating the p53 pathway (Zhang et al., 2021d). The relationship between SPI1, MBD1, and diabetic nephropathy needs further experimental verification. According to the literature, mir-33a regulates insulin signaling and fatty acid metabolism and plays a role in the development of diabetes and its complications (Dávalos et al., 2011; Nikpour et al., 2014). According to another study, C1 is regulated by LEF1 and has-mir-33a (Wang et al., 2016) and is involved in the development of DN. Recent studies have shown that dapagliflozin acts as a nephroprotective agent for DN by counteracting hsa_circRNA_012448-has-miR-29b-2-5p-GSK3β pathway-mediated oxidative stress (Song et al., 2022), and has-miR-29b-2-5p expression was also screened in our mRNA-miRNA network. The PubMed literature search showed that the relevance of our screened miRNAs such as miR-29b-2-5p, miR-34a-5p, miR-27a-3p, miR-146a-5p, miR-155-5p (Cao et al., 2022), miR-103a-3p (Jing et al., 2022) and miR-103a-3pto DN has been confirmed by research and that more signaling pathways remain to be further investigated. has-miR-34a-5p has been shown to be a salient biomarker of diabetes, involved in oxidative stress (Banerjee et al., 2017), vascular senescence (Ito et al., 2010). It was found that the expression of has-miR-34a-5p was associated with LAPTM5 in DN. Among the regulatory networks constructed, has-miR-34a-5p, has-miR-27a-3p, and has-miR-146a-5p were found as molecules coordinating the regulation of hub genes. Gholaminejad et al. (2021) identified miR-34a-5p, miR-129-2-3p, and miR-27a-3p as the top regulatory molecules produced in immunoglobulin A nephropathy. Has-miR-146a-5p and has-miR-30a-5p expression levels were suggested by Baker et al. (2017) for the identification of DN and renal diseases other than IgA nephropathy.

The current study discusses eight potentially key genes in the development of diabetic nephropathy as potential mechanisms involved in diabetic nephropathy. The genes might be prospective biomarkers and treatment goals for diabetic nephropathy. Also, there are some limitations in this paper: the dataset samples included (age, cells, race, lifestyle, and family history) may affect the stability of the results. The analysis of key potential molecules gained from this study needs to be further validated in the clinical trial.

## Conclusion

Our study explored the same platform GEO dataset for diabetic nephropathy bioinformatics analysis and identified eight potential key genes (TYROBP, ITGB2, CD53, IL10RA, LAPTM5, CD48, C1QA, and IRF8), screened eight transcription factors, and identified 93 miRNA nodes. It provides some contribution to identifying new biomarkers of diabetic nephropathy susceptibility and useful potential targets for therapy.

## Data Availability

The original contributions presented in the study are included in the article/supplementary material, further inquiries can be directed to the corresponding author.
